# Supplementary material for: Incentive salience attribution, “sensation-seeking” and “novelty-seeking” are independent traits in a large sample of male and female heterogeneous stock rats
Source: Sci Rep. 2019 Feb 20;9:2351. doi: 10.1038/s41598-019-39519-1 (PMC6382850; doi:10.1038/s41598-019-39519-1)
Supplement: Supplementary file 1 — Supplementary Information [file 41598_2019_39519_MOESM1_ESM.pdf]

Supplementary Information

Incentive salience attribution, "sensation-seeking" and "novelty-seeking" are independent traits in a large sample of male and female heterogeneous stock rats

Alesa R. Hughson[1], Aidan P. Horvath[1], Katie Holl[2], Abraham A. Palmer[3], Leah C. Solberg Woods[4], Terry E. Robinson[1], Shelly B. Flagel[5-6]*

[1]Department of Psychology, University of Michigan, Ann Arbor, USA
[2]Department of Medicine, Medical College of Wisconsin, Milwaukee, USA
[3]Department of Psychiatry, University of California San Diego, La Jolla, USA
[4]Department of Internal Medicine, Molecular Medicine, Center on Diabetes, Obesity and Metabolism, Wake Forest School of Medicine, Winston-Salem, USA
[5]Department of Psychiatry, University of Michigan, Ann Arbor, USA
[6]Molecular and Behavioral Neuroscience Institute, University of Michigan, Ann Arbor, USA
*sflagel@umich.edu

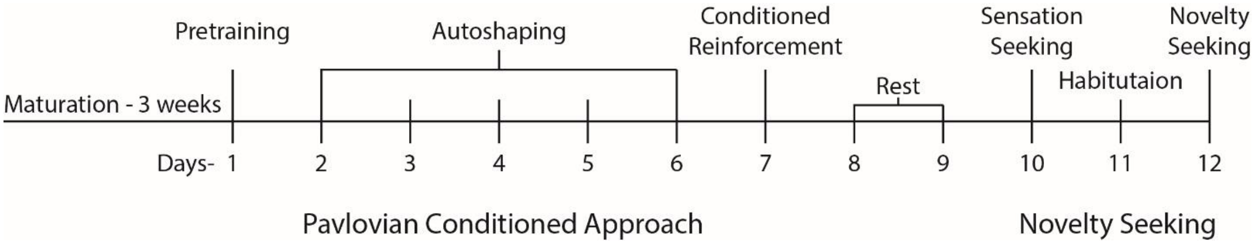

Supplemental Figure 1. *Experimental timeline.* Timeline of experiment from rats' arrival to the end of the novelty-seeking test. Rats arrived ~35 days of age and were allowed ~3 weeks to acclimate and mature to adulthood. Behavioral testing commenced when they were ~60 days old and was completed by the time they were ~75 days old.

Supplemental Table 1

	Full Sample		Female		Male		GT		IN		ST	
**Behavioral Variables**	Factor1	Factor2	Factor1	Factor2	Factor1	Factor2	Factor1	Factor2	Factor1	Factor2	Factor1	Factor2
PCA Index Score	**0.803**		**0.785**		**0.822**			**0.708**	**0.706**		0.653	0.396
Incentive Value Index	**0.821**		**0.817**		**0.821**		**0.771**		**0.726**		**0.790**	
Sensation Seeking	0.435		0.407		0.372		**0.755**		0.480	-0.386	0.409	
Novelty Seeking		**0.981**		**0.971**		**0.976**		**0.733**		**0.925**		**0.918**

	GT Female		GT Male		IN Female		IN Male		ST Female			ST Male
**Behavioral Variables**	Factor1	Factor2	Factor1	Factor2	Factor1	Factor2	Factor1	Factor2	Factor1	Factor2	Factor3	Factor1
PCA Index Score	0.462	0.505		**0.783**	0.601		**0.737**		**0.786**			0.688
Incentive Value Index	**0.768**		**0.766**		**0.744**	0.328	**0.781**		**0.774**		0.302	**0.710**
Sensation Seeking	**0.733**		0.660		0.535	-0.325		**0.907**			**0.952**	0.385
Novelty Seeking		**0.888**		0.615		**0.895**		0.415		**0.948**		-0.364

Supplemental Table 1. Principal Components Analysis. Factor loadings from the rotated component matrix for the entire population (i.e. full sample) and for each sex and phenotype considered separately.
